# Deletional tolerance prevents AQP4‐directed autoimmunity in mice

**DOI:** 10.1002/eji.201646855

**Published:** 2017-01-25

**Authors:** Anna‐Lena Vogel, Benjamin Knier, Katja Lammens, Sudhakar Reddy Kalluri, Tanja Kuhlmann, Jeffrey L. Bennett, Thomas Korn

**Affiliations:** ^1^ Klinikum rechts der Isar, Department of NeurologyTechnical University of MunichMunichGermany; ^2^ Klinikum rechts der Isar, Department of Experimental NeuroimmunologyTechnical University of MunichMunichGermany; ^3^ Department of Biochemistry at the Gene CenterLudwig‐Maximilians‐UniversityMunichGermany; ^4^ Institute of NeuropathologyUniversity Hospital MünsterMünsterGermany; ^5^ Department of NeurologySchool of MedicineUniversity of ColoradoAuroraCOUSA; ^6^ Department of OphthalmologySchool of MedicineUniversity of ColoradoAuroraCOUSA; ^7^ Program in NeuroscienceSchool of MedicineUniversity of ColoradoAuroraCOUSA; ^8^ Munich Cluster for Systems Neurology (SyNergy)MunichGermany

**Keywords:** Aquaporin 4 (AQP4), B cell, Encephalitogenic epitope, Experimental neuromyelitis optica, Optical coherence tomography, T cell, Tolerance

## Abstract

Neuromyelitis optica (NMO) is an autoimmune disorder of the central nervous system (CNS) mediated by antibodies to the water channel protein AQP4 expressed in astrocytes. The contribution of AQP4‐specific T cells to the class switch recombination of pathogenic AQP4‐specific antibodies and the inflammation of the blood–brain barrier is incompletely understood, as immunogenic naturally processed T‐cell epitopes of AQP4 are unknown. By immunizing *Aqp4* ^−/−^ mice with full‐length murine AQP4 protein followed by recall with overlapping peptides, we here identify AQP4(201‐220) as the major immunogenic IA^b^‐restricted epitope of AQP4. We show that WT mice do not harbor AQP4(201–220)‐specific T‐cell clones in their natural repertoire due to deletional tolerance. However, immunization with AQP4(201–220) of *Rag1* ^−/−^ mice reconstituted with the mature T‐cell repertoire of *Aqp4* ^−/−^ mice elicits an encephalomyelitic syndrome. Similarly to the T‐cell repertoire, the B‐cell repertoire of WT mice is “purged” of AQP4‐specific B cells, and robust serum responses to AQP4 are only mounted in *Aqp4* ^−/−^ mice. While AQP4(201–220)‐specific T cells alone induce encephalomyelitis, NMO‐specific lesional patterns in the CNS and the retina only occur in the additional presence of anti‐AQP4 antibodies. Thus, failure of deletional T‐cell and B‐cell tolerance against AQP4 is a prerequisite for clinically manifest NMO.

## Introduction

Neuromyelitis optica (NMO) is an autoimmune inflammatory disease of the central nervous system (CNS). Unlike in multiple sclerosis, the autoreactive adaptive immune response in NMO is directed against a known target antigen, namely the water channel protein aquaporin 4 (AQP4). AQP4 is characterized by six transmembrane helices and is expressed in astrocytic end feet of the glia limitans 1. However, AQP4 is not only expressed in the CNS but also in Müller cells of the retina, in stomach, kidney, and skeletal muscle 2. Thus, AQP4 is not sequestered behind the blood–brain barrier. In contrast, the expression of other CNS autoantigens including myelin oligodendrocyte glycoprotein (MOG) is restricted to the CNS, and it is believed that the adaptive immune system ignores MOG due to its “sequestered” expression in the CNS, although MOG‐reactive T‐cell clones are readily detected in the normal T‐cell repertoire of wild type (WT) mice 3. However, due to its wide expression outside the CNS, antigenic “ignorance” is likely not a tolerance mechanism in the case of AQP4.

It is clear that anti‐AQP4 antibodies that target astrocytic AQP4 molecules are necessary for the pathologic process in NMO patients 4, 5. The mechanism of anti‐AQP4‐induced damage has been resolved to some extent, and two major effector functions of anti‐AQP4 antibody binding to its target have been proposed: First, internalization of the M1 isoform of AQP4 with functional consequences for other proteins including the glutamate transporter EAAT2 that are associated with the M1 isoform of AQP4 6, 7, and second, anti‐AQP4 antibody binding to the M23 isoform of AQP4 that forms large orthogonal arrays of particles that resist internalization but are targeted for complement‐mediated lysis upon binding of anti‐AQP4 antibodies 8, 9, 10, 11, 12. Here, the presence of complement regulators including CD46 (membrane cofactor protein), CD55 (decay accelerating factor), and CD59 (protectin) in close proximity to AQP4 in peripheral tissues but their absence in the CNS has been proposed to explain why astrocytes, but not peripheral epithelial cells or parenchymal cells, are particularly susceptible to complement‐mediated lysis in patients with high serum titers of anti‐AQP4 antibodies 13. While the AQP4‐specific B‐cell response and also the pattern of CNS damage resulting from antibody‐mediated immunopathology have been well characterized and translated into diagnostic criteria 14 and therapeutic approaches 15, 16, 17, little is known about the underlying AQP4‐specific T‐cell response. Yet, a potent AQP4‐specific T‐cell response must be assumed in patients with NMO because anti‐AQP4 antibodies are class‐switched complement binding antibodies that need T‐cell help in order to be generated 18, 19. Moreover, certain HLA haplotypes (DR3) are overrepresented in NMO patients 20, 21, 22. However, it is unknown how T‐cell tolerance against the widely expressed antigen AQP4 is broken and whether antigen‐specific T cells are required for the induction of antibody‐mediated immunopathology at the gliovascular interface of the CNS.

Here, we found that the major encephalitogenic T‐cell epitope of AQP4 is tightly thymically tolerized in WT mice. Similarly, the WT B‐cell repertoire is essentially devoid of AQP4‐specific B cells. We have identified and fine mapped the major T‐cell epitope of AQP4 and observed that in a transfer model, AQP4‐specific T cells alone induced an encephalomyelitic syndrome. However, only in the additional presence of anti‐AQP4 antibodies, CNS lesions are reminiscent of NMO. Moreover, retinal pathology, which has been described in NMO, appears to be dependent on anti‐AQP4 antibodies while AQP4‐specific T cells alone failed to induce retinal changes as measured by optical coherence tomography (OCT). Thus, understanding how T‐ and B‐cell tolerances to AQP4 are broken is key in identifying causal treatment options for NMO.

## Results

### IA^b^‐restricted immunogenic epitopes of AQP4

**Figure 1:**
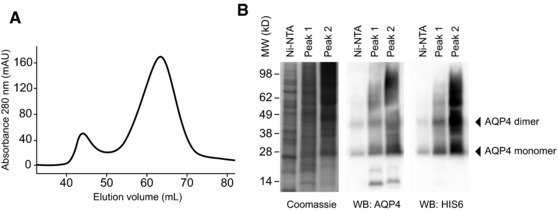
Protein expression and purification. The full‐length AQP4 isoform M1 was produced in High Five cells using the Baculovirus‐insect cell expression system. (**A**) Purification of the protein by ultracentrifugation, Ni‐NTA affinity chromatography, and subsequent gel filtration on a S200 HR 16/60 column. n‐Octyl‐β‐d‐glucopyranoside was used as detergent throughout the process. (**B**) Side‐by‐side analysis of the Ni‐NTA elution fraction as well as the pooled fractions of gel filtration peaks 1 and 2 using Coomassie staining and western blot analysis for detection of AQP4 and HIS6. Data are representative of six independent experiments (**A, B**).

**Figure 2:**
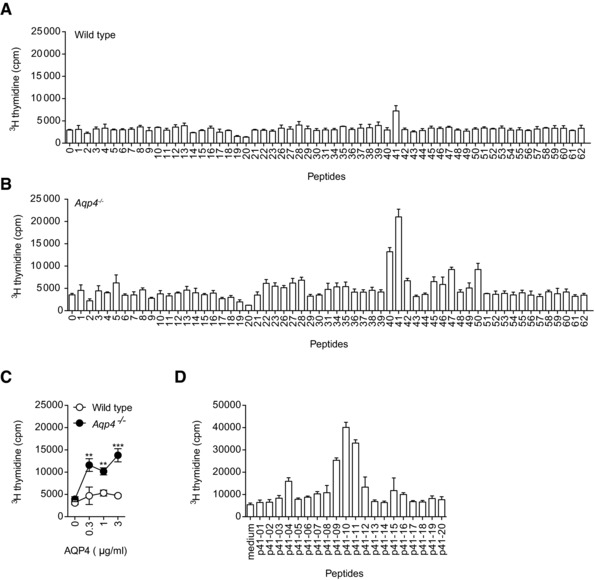
AQP4(201–220) is the immunodominant IA^b^‐restricted epitope of AQP4. WT C57BL/6 and *Aqp4* ^−/−^ mice were immunized s.c. with full‐length AQP4 protein (**A**, **B**, and **D**) or AQP4(201–220) peptide (**C**) emulsified in CFA. Draining LN cells and splenocytes were analyzed for cell proliferation in response to single peptides or full‐length AQP4 protein. (**A, B**) Splenocytes and LN cells from AQP4 immunized WT and *Aqp4* ^−/−^ mice were stimulated with single 20‐mer peptides spanning the whole sequence of AQP4. (**C**) Draining LN cells of AQP4(201–220) immunized WT and *Aqp4* ^−/−^ mice were tested for their proliferative recall response to full‐length AQP4 protein. (**D**) Fine mapping of the immunodominant AQP4 epitope was performed using 11‐mer peptides spanning AQP4(196–225) to recall cells from AQP4‐immunized *Aqp4* ^−/−^ mice. Proliferation was measured by ^3^[H]‐thymidine incorporation. Means of triplicate cultures ± SD are shown. **p < 0.01, ***p < 0.001 (Student's *t*‐test). Data are representative of five independent experiments (**A–D**).

AQP4 is a transmembrane protein. In order to have a reliable source of AQP4 protein antigen, we expressed the full‐length M1 isoform of mouse AQP4 with a C‐terminal His tag in a Baculovirus system (Fig. 1). We have previously reported that AQP4(22–36) was an IA^b^‐restricted epitope of AQP4 23. However, AQP4(22–36)‐specific T cells did not respond to full‐length AQP4 protein, and immunization of WT animals with AQP4(22–36) failed to induce any immunopathology in the CNS, suggesting that AQP4(22–36) was no naturally processed epitope 23. In order to assess the entire AQP4 protein for possible encephalitogenic epitopes in a situation without deletional T‐cell tolerance against AQP4, we immunized *Aqp4* ^−/−^ mice 24 with AQP4 protein emulsified in complete Freund's adjuvant (CFA), followed by recall assays of draining LNs and spleen with AQP4 peptide 20‐mers spanning the entire mouse AQP4 sequence and overlapping by 15 amino acids. *Aqp4* ^−/−^ mice raised a strong proliferative T‐cell response against AQP4 with an immunogenic epitope in the region of AQP4(196–225), while T cells from AQP4 immunized WT mice only showed a weak reactivity against AQP4 (Fig. 2A and B). Thus, the major immunogenic epitope AQP4(201–220) appeared to be tolerized in WT mice.

Next, we tested whether AQP4(201–220) (p41) was a naturally occurring epitope of AQP4. *Aqp4* ^−/−^ mice were immunized with AQP4(201–220), and draining LN cells were interrogated for their proliferative response to full‐length AQP4 protein (Fig. 2C). Since AQP4 is a transmembrane protein with limited solubility, only low concentrations were added to the recall culture. Yet, AQP4(201–220) responsive T cells also responded to the entire AQP4 protein, suggesting that processing of AQP4 by antigen presenting cells (APCs) resulted in epitopes similar or identical to AQP4(201–220). The fine mapping of this region revealed that AQP4(205–215) is the core epitope (Fig. 2D). Taken together, AQP4(201–220) is the major immunogenic T‐cell epitope of AQP4 and appears to be efficiently tolerized in AQP4‐sufficient mice.

### *Aqp4* ^−/−^ mice raise a potent serum response against AQP4

**Figure 3:**
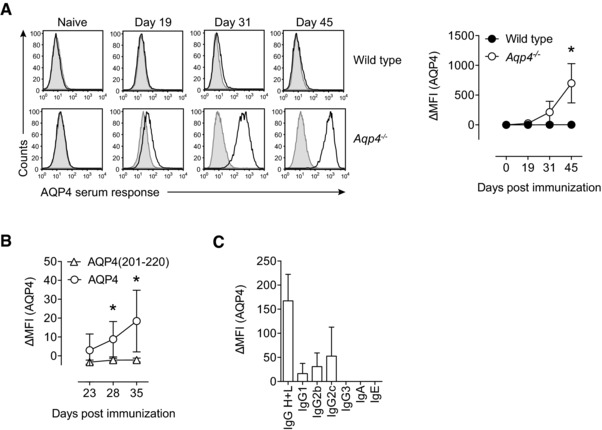
*Aqp4* ^−/−^ mice but not WT controls mount a robust antibody response to AQP4 upon immunization with full‐length AQP4 protein. WT C57BL/6 and *Aqp4* ^−/−^ mice were immunized s.c. with full‐length AQP4 protein or AQP4(201–220) peptide emulsified in CFA. (**A**) Sera of naive mice and AQP4‐immunized WT or *Aqp4* ^−/−^ mice at different time points after immunization with AQP4 were analyzed for AQP4‐specific antibodies in a cell‐based flow cytometric assay with LN18 cells transduced with AQP4 expressing lentivirus. Anti‐mouse total IgG H+L (AlexaFluor488 labeled) was used to detect anti‐AQP4 antibodies (black line histograms). The ΔMFI was calculated in relation to staining of LN18 cells transduced with empty vector (shaded histograms). Representative histograms (left) and plot of ΔMFI (right). Mean ΔMFI ± SD (*n* = 6 per group). (**B**) AQP4 serum response at different time points in *Aqp4* ^−/−^ mice that were immunized with AQP4 protein or AQP4 (201–220) peptide. Mean ΔMFI ± SD (*n* = 6 per group). (C) To specify the antibody classes and subclasses, fluorochrome‐labeled anti‐mouse Ig antibodies specific for IgA, IgE, IgG1, IgG2b, IgG2c, and IgG3 were used. Mean ΔMFI ± SD (*n* = 6). *p < 0.05 (unpaired Student's *t*‐test). Data are representative of three independent experiments (**A**–**C**).

Since NMO is an antibody‐mediated autoimmune disease of the CNS, we next studied the antigen‐specific serum response upon immunization with AQP4 protein. While immunization with AQP4 in CFA did not elicit an antibody response to AQP4 in WT mice, *Aqp4* ^−/−^ mice raised a robust anti‐AQP4 serum response as measured by a cell‐based assay 25 (Fig. 3A, Supporting Information Fig. 1). We chose this cell‐based assay in order to measure conformational antibodies of potential pathogenic relevance. Notably, only immunization with full‐length AQP4 protein, but not immunization with the immunodominant T‐cell epitope AQP4(201–220), resulted in the generation of antibodies that recognized the natural conformation of AQP4 (Fig. 3B). Anti‐AQP4 antibodies were predominantly of the IgG2c isotype, and thus complement fixing, which suggested that they had the potential to induce immunopathology when binding to their target antigen (Fig. 3C).

### Adaptive anti‐AQP4 responses are prevented by deletional tolerance

Failure to raise a proliferative anti‐AQP4 T‐cell recall response in WT mice upon immunization with AQP4 protein suggested that the precursor frequency of AQP4‐specific T cells in the physiologic repertoire was extremely low. Deletional tolerance (negative selection) leads to purging of the mature T‐cell repertoire of clones reactive to tissue‐restricted antigens 26. In order to narrow down the mechanism of tolerance induction to AQP4, we took complementary approaches as follows.

**Figure 4:**
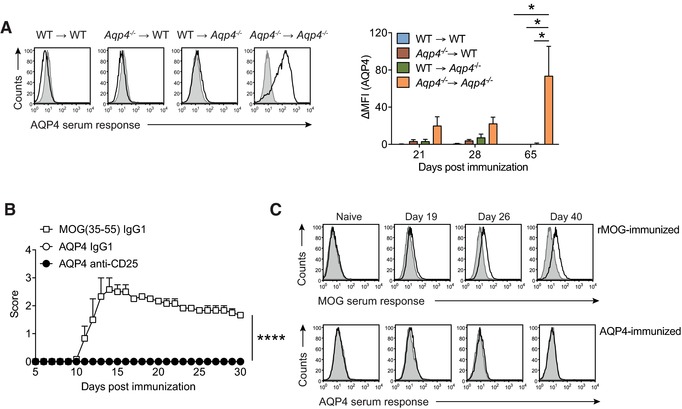
The naive T‐cell and B‐cell repertoires of WT mice are purged of AQP4 reactive clones. (**A**) Criss‐cross BM chimeras of WT C57BL/6 and *Aqp4* ^−/−^ mice were immunized with full‐length AQP4 protein emulsified in CFA. Sera from different time points after immunization were tested for AQP4‐specific antibodies in a cell‐based flow cytometric assay as described in Fig. 3. Representative histograms (left) and Mean ΔMFI ± SD (*n* = 6 per group) for AQP4‐specific IgG (right). *p < 0.05 (ANOVA plus Sidak's post‐test). (**B**) WT C57BL/6 mice were immunized s.c. with full‐length AQP4 protein or MOG(35–55) emulsified in CFA as immunization control. On days –5 and –3 prior to immunization, some mice were injected i.p. with control IgG1 or with anti‐CD25 antibodies to deplete T_reg_ cells before immunization with full‐length AQP4 protein. Mean clinical scores ± SEM (*n* = 6 per group). ****p < 0.0001 (Mann–Whitney *U* test for nonparametric values). (**C**) *Aqp4* ^ΔT^ x *Tcra* ^−/−^ mice were generated by i.p. transfer of the mature T‐cell compartment of *Aqp4* ^−/−^ mice into *Tcra* ^−/−^ mice. The mice were immunized with full‐length rMOG or AQP4 protein emulsified in CFA. Sera of mice from each group were collected at different time points after immunization and tested for MOG‐ and AQP4‐specific antibodies in a cell‐based flow cytometric assay as described. Representative histogram plots illustrating the anti‐MOG and anti‐AQP4 serum responses in *Aqp4* ^ΔT^ × *Tcra* ^−/−^ mice at different time points (*n* = 6 per group). Data are representative of two independent experiments (**C**).

First, AQP4 is an Aire‐dependent gene 27, and besides expression in mTECs, tolerance to Aire‐dependent genes might be induced in the thymus by hematopoietic APCs, in particular by dendritic cells, which can cross‐present Aire‐dependent genes 28, 29, and more importantly also by B cells, a subset of which express Aire themselves 30. Thus, transplantation of *Aqp4* ^−/−^ bone marrow (BM) into WT recipients should rescue AQP4‐specific T‐cell reactivities during shaping of the T‐cell repertoire if negative selection of AQP4‐specific thymocytes was mediated by hematopoietic cells presenting their intrinsic AQP4 in the thymus (e.g., thymic B cells). However, we did not measure AQP4‐specific T‐cell and B‐cell responses in *Aqp4* ^−/−^ → WT BM chimeras upon immunization with full‐length AQP4 protein and only detected sizeable AQP4‐specific antibody responses in *Aqp4* ^−/−^ → *Aqp4* ^−/−^ controls (Fig. 4A), suggesting that deletional tolerance to AQP4 was “classically” generated by expression of AQP4 in radiation‐resistant thymic stromal cells.

Second, besides deletion, an alternative fate of AQP4‐specific thymocytes, whose T‐cell receptor (TCR) is engaged by its cognate ligand, is deviation into the Foxp3^+^ regulatory T (T_reg_) cell lineage 31. Since the frequency of antigen‐specific Foxp3^+^ T_reg_ cells is difficult to measure without MHC class II tetramers, we decided to take a functional approach and deleted Foxp3^+^ T_reg_ cells in the peripheral immune compartment using an antibody to CD25. This method is highly efficient in breaking tolerance mediated by increased T_reg_‐cell frequencies 32. Yet, immunization of T_reg_‐cell depleted WT mice with AQP4 protein failed to induce clinical signs of encephalomyelitis (Fig. 4B). No other signs of disease were observed in these mice either. Thus, T_reg_‐cell depletion did not break T‐cell tolerance to AQP4, suggesting that dominant peripheral tolerance was not responsible for the failure of WT mice to mount AQP4‐specific encephalitogenic T‐cell responses.

Third, we assessed the AQP4‐specific B‐cell response separately. For this purpose, we transferred the mature (nontolerized) CD4^+^ T‐cell compartment from *Aqp4* ^−/−^ mice, which carried sufficiently high AQP4‐specific T‐cell precursor frequencies to mount robust AQP4‐specific T‐cell recall responses (Fig. 2), into *Tcra* ^−/−^ mice that lack endogenous T cells but have an unaltered mature B‐cell repertoire. We termed the reconstituted animals *Aqp4* ^ΔT^ × *Tcra* ^−/−^ mice. We immunized these mice with AQP4 protein or with the alternative CNS antigen MOG protein, for which deletional tolerance is known to be unimportant 3, and tested the serum response to AQP4 protein or MOG protein, respectively, using a cell‐based assay. While *Aqp4* ^ΔT^ × *Tcra* ^−/−^ mice mounted a clear anti‐MOG antibody response upon immunization with MOG protein, they failed to generate anti‐AQP4 antibodies (Fig. 4C), despite measurable proliferative T‐cell responses to recall with AQP4 protein or AQP4(201–220) (data not shown). Thus, in addition to tight thymic T‐cell tolerance, the endogenous B‐cell repertoire appears to be devoid of AQP4‐reactive B cells when AQP4 is normally expressed in peripheral tissues. In summary, both the T‐ and B‐cell repertoires are efficiently purged of AQP4 reactive clones under physiologic conditions.

### AQP4‐specific T‐cell responses alone induce an encephalomyelitic syndrome

**Figure 5:**
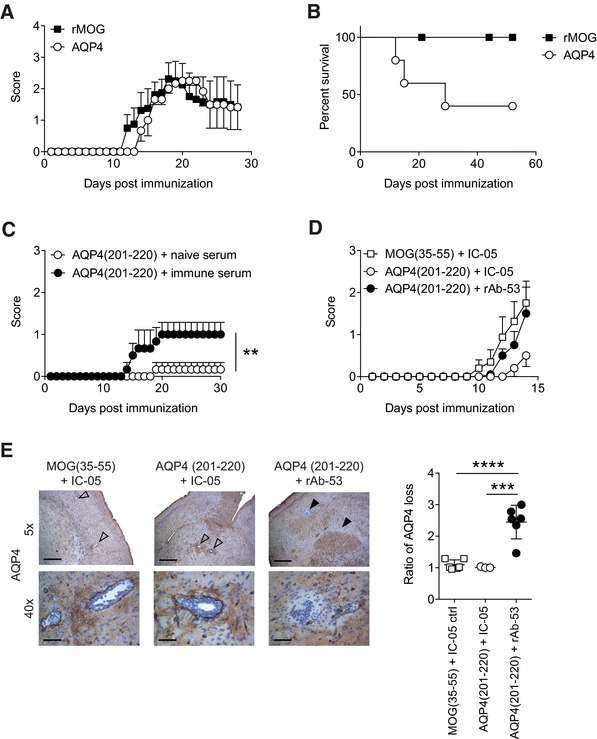
AQP4(201–220) is an encephalitogenic epitope. (**A, B**) *Aqp4* ^ΔT^ × *Tcra* ^−/–^ mice were immunized with full‐length AQP4 or rMOG protein emulsified in CFA and monitored for symptoms of encephalomyelitis. (**A**) Mean clinical scores ± SEM and (**B**) percent survival of immunized mice are shown (*n* = 6 per group). (**C**) *Aqp4* ^ΔT^ × *Rag1* ^−/−^ mice were immunized with AQP4(201–220) peptide emulsified in CFA. One hundred microliters of naïve serum or immune serum with high titers of anti‐AQP4 antibodies (harvested from AQP4‐immunized *Aqp4* ^−/−^ mice) were administered i.v. on days 6 and 12 after immunization. Mean clinical scores ± SEM (*n* = 5 per group). **p < 0.01 (Mann–Whitney *U* test for nonparametric values). (**D, E**) *Aqp4* ^ΔT^ × *Rag1* ^−/−^ mice were immunized with either AQP4(201–220) or MOG(35–55) peptide emulsified in CFA. On days 12 and 14 after immunization, the mice were injected i.v. with rAb‐53 antibody or IC‐05 control antibody. (**D**) Mean clinical scores ± SEM (*n* = 6 per group). (**E**) Mice were sacrificed 4 h after the last antibody treatment to perform histological analysis. Representative AQP4 staining of the brain at 5× (scale bar, 400 μm) and 40× magnification (scale bar, 50 μm). Open arrows show vessels without perivascular loss of AQP4 immunoreactivity. Closed arrows indicate areas of AQP4 loss in the vicinity of vessels. Quantification of AQP4 loss as ratio of the area with AQP4 signal loss divided by the area of the associated vessel lumen in the brain of the indicated treatment groups (mean ± SD). ***p < 0.001; ****p < 0.0001 (ANOVA plus Sidak's post‐test). Data are representative of two independent experiments (**A**, **B**, **D**, and **E**).

Despite the failure to raise an AQP4‐specific serum response, *Aqp4* ^ΔT^ × *Tcra* ^−/−^ mice developed a neurologic syndrome, which was clinically similar to the disease exhibited by *Aqp4* ^ΔT^ × *Tcra* ^−/−^ mice that were immunized with MOG protein (Fig. 5A). AQP4‐immunized *Aqp4* ^ΔT^ × *Tcra* ^−/−^ mice developed paresis of the tail and the hind limbs but notably, tended to have a higher mortality than MOG protein immunized *Aqp4* ^ΔT^ × *Tcra* ^−/−^ mice (Fig. 5B).

**Figure 6:**
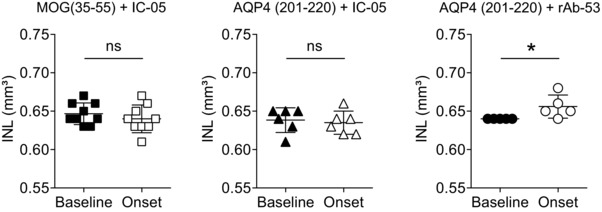
*Aqp4* ^ΔT^ × *Rag1* ^−/−^ mice immunized with AQP4(201–220) peptide plus i.v. application of rAb‐53 develop inner nuclear layer (INL) swelling at disease onset. *Aqp4* ^ΔT^ × *Rag1* ^−/−^ mice were immunized with either AQP4(201–220) or MOG(35–55) peptide emulsified in CFA. After immunization, the mice were injected i.v. with rAb‐53 antibody or IC‐05 control antibody on day 12 after immunization. Retinal optical coherence tomography (OCT) was performed in the indicated treatment groups prior to immunization (baseline) and on day 13 after immunization at the onset of disease (and 1 day after administration of control antibody IC‐05 or anti‐AQP4 antibody rAb‐53). Mean INL volumes of individual eyes ± SD are shown. *p < 0.05, ns = not significant (Student's *t*‐test). One experiment with six mice per group is shown.

Next, we wanted to test whether AQP4(201–220)‐specific T cells alone were sufficient to induce encephalomyelitis in mice. Because AQP4(201–220)‐specific T‐cell responses were only elicited in *Aqp4* ^−/−^ mice that lack the target of the immune response, we had to again construct compound mice. We transferred the mature CD4^+^ T‐cell repertoire of *Aqp4* ^−/−^ mice into *Rag1* ^−/−^ recipients (*Aqp4* ^ΔT^ × *Rag1* ^−/−^ mice) and immunized these mice with AQP4(201–220) peptide in CFA. AQP4(201–220)‐specific T cells were capable of inducing minor signs of disease (Fig. 5C). The disease severity was significantly aggravated when in addition to inducing AQP4(201–220)‐specific T cells, AQP4 immune serum generated in *Aqp4* ^−/−^ mice was administered into *Aqp4* ^ΔT^ × *Rag1* ^−/−^ mice (Fig. 5C). Similarly, intravenous (i.v.) transfer of a monoclonal antibody to AQP4 (rAb‐53) in a murinized framework aggravated the disease phenotype of *Aqp4* ^ΔT^ × *Rag1* ^−/−^ mice immunized with AQP4(201–220) peptide as compared with administration of an irrelevant control antibody (IC‐05) (Fig. 5D). Despite similar amounts of T‐cell infiltrates and infiltrates of macrophages (data not shown), perivascular loss of immunoreactivity against AQP4 in the CNS was only observed in AQP4(201–220)‐immunized *Aqp4* ^ΔT^ × *Rag1* ^−/−^ mice that had received anti‐AQP4 antibodies but not in AQP4(201–220)‐immunized mice treated with nonimmune serum or irrelevant antibodies (Fig. 5E). Finally, we performed retinal OCT. Here, retinal Müller cells, which are located in the inner nuclear layer (INL), express high amounts of AQP4 and represent a primary target for both AQP4‐specific antibodies and T‐cell‐driven retinitis 33. As compared to baseline (prior to immunization), INL volumes remained unchanged in *Aqp4* ^ΔT^ × *Rag1* ^−/−^ mice immunized with MOG(35–55) or AQP4(201–220), which received a control antibody. In contrast, a significant increase in INL volumes was detected when AQP4(201–220) immunized *Aqp4* ^ΔT^ × *Rag1* ^−/−^ mice were treated with rAb‐53 (Fig. 6). Thus, T‐cell‐mediated retinitis alone is not sufficient to induce an increase in INL volume while anti‐AQP4 antibodies in the presence of a T‐cell‐mediated retinitis result in INL swelling.

Taken together, AQP4(201–220)‐specific T cells alone induce a clinically manifest encephalomyelitis. However, NMO‐specific lesional patterns in the CNS are only achieved in the additional presence of anti‐AQP4 antibodies.

## Discussion

In the present study, we identify AQP4(201–220) as the major MHC class II (IA^b^)‐restricted immunogenic epitope of AQP4. We show that this epitope is naturally processed, and in principle, elicits an encephalitogenic T‐cell response. However, TCRs recognizing AQP4(201–220) are efficiently deleted from the mature T‐cell repertoire by thymic negative selection. Similarly, the B‐cell repertoire of WT mice is essentially devoid of AQP4‐specific B‐cell receptors (BCRs) because reconstitution of AQP4‐reactive T cells fails to rescue an anti‐AQP4 serum response in a WT B‐cell repertoire while robust anti‐AQP4 serum responses are mounted in *Aqp4* ^−/−^ mice whose B‐cell repertoire is educated in the absence of AQP4. Despite the encephalitogenic potential of AQP4(201–220)‐specific T cells, NMO‐like lesions in the CNS, which are characterized by perivascular loss of AQP4 immunoreactivity, are only induced in the presence of AQP4(201–220)‐specific T cells and anti‐AQP4 antibodies but not by AQP4(201–220)‐specific T cells alone. Thus, we propose that both thymic tolerance and B‐cell tolerance need to fail in order to set the stage for NMO‐like autoimmune pathology in the CNS.

The significance of an AQP4‐specific T‐cell response in experimental NMO, and by analogy also in NMO patients, has been discussed in the context of T‐cell help required to generate pathogenic anti‐AQP4 antibodies 34 and in the context of the potential of AQP4‐specific T cells to induce an inflammatory milieu at the blood–brain barrier, which may be the prerequisite for anti‐AQP4 antibodies to efficiently reach their target in astrocytic end feet and trigger complement‐mediated tissue destruction 35. Since heterologous systems and in particular the transfer of human anti‐AQP4 IgG plus human complement into mice has certain disadvantages 36, we here used a completely homologous murine system. Early work suggested that a mild EAE induced by adoptive transfer of encephalitogenic T cells of unrelated specificity (against myelin basic protein) is required to enable access of i.v. transferred human anti‐AQP4 antibodies to their target in the rat CNS 11, 12. Thus, any T‐cell‐mediated inflammation of the blood–brain barrier appeared to be sufficient to enable anti‐AQP4 antibody mediated tissue damage. However, a more meticulous investigation of the effector function of AQP4‐specific T cells in situ has recently suggested that AQP4‐specific T cells may determine the predilection sites that are typically affected by anti‐AQP4 antibody mediated pathology 35. In that study, AQP4 peptides predicted to be good binders to the Lewis rat specific MHC class II complex (RT1.B^L^) were used to immunize rats. While no clinical disease was induced by active immunization with AQP4 peptides, RT1.B^L^‐restricted AQP4(268–285)‐specific T‐cell lines were generated and induced an encephalomyelitic syndrome upon in vitro activation and transfer into naive recipient rats. However, only the additional transfer of heterologous human anti‐AQP4 IgG produced NMO‐like lesions at NMO predilection sites, in particular the spinal cord gray matter 35. Using the same model, damage to the retina has been identified as a primary event (and not secondary to optic neuritis) in experimental NMO 33. Here, retinitis is induced by AQP4(268–285)‐specific T cells alone. However, loss of AQP4 immunoreactivity in Müller cell side branches in the INL (but not the ganglion cell layer) is only observed in the presence of both AQP4‐specific T cells and anti‐AQP4 antibodies 33. Our finding that INL swelling as measured by retinal OCT only occurred in the presence of both AQP4‐specific T cells and anti‐AQP4 antibodies but not in T‐cell‐mediated encephalomyelitis with either AQP4 or MOG as target antigens alone is in line with a loss‐of‐function of Müller cells, which play an important role in water and potassium homeostasis of the INL of the retina 37.

While the effector phase of NMO is increasingly well understood and major advances in the field have been reviewed 1, the events that trigger adaptive immune responses to AQP4 are not known. Recently, a *Clostridium perfringens* antigen has been identified to be a molecular mimic, which might activate HLA‐DR‐restricted AQP4(61–80)‐specific T cells in humans 38. Although the authors of that study suggested that AQP4(61–80) is a naturally processed determinant of AQP4, it is unclear whether AQP4(61–80)‐specific T cells contribute to the formation of anti‐AQP4 antibodies. Furthermore, T‐cell epitopes of AQP4 have been reported in rats 39, mice 23, 40, 41, and humanized DRB1*0301 transgenic mice 42, but none of them has been confirmed to be a naturally processed epitope since active immunization has never been reported to induce clinical signs of disease in any of these models. Besides AQP4(201–220), a further IA^b^‐restricted epitope of AQP4, i.e. AQP4(135–153), has been reported in the repertoire of *Aqp4* ^−/−^ mice 41, 43. However, since AQP4(135–153) was inferred as a relevant epitope by using an “in silico” approach predicting binding affinities of peptides to IA^b^ 43, it is possible that AQP4(135–153) may not be a naturally processed epitope of AQP4. Here, we did not find a significant AQP4(135–153)‐specific recall response upon immunization of *Aqp4* ^−/−^ mice with full‐length murine AQP4 protein. However, we show that IA^b^‐restricted AQP4(201–220) is indeed a naturally processed epitope of AQP4 and is able to induce an encephalomyelitic syndrome upon active immunization of *Aqp4* ^ΔT^ × *Rag1* ^−/−^ mice.

In the present study, we provide indirect evidence that the B‐cell repertoire of WT mice is essentially devoid of AQP4 reactive BCRs. We conclude this from our finding that despite sufficient antigen‐specific T‐cell help, no antigen‐specific serum response was raised within the natural B‐cell repertoire. In contrast, robust anti‐AQP4 antibody titers were generated in *Aqp4* ^−/−^ mice. Thus, B cells with AQP4‐specific BCRs are most likely depleted of the WT BCR repertoire in an antigen‐specific manner. In fact, AQP4 is almost ubiquitously expressed (for review, see 2) and apoptotic AQP4‐expressing cells might lead to physical or “functional” purging of the B‐cell repertoire, for example, by receptor editing, anergy, or activation‐induced cell death, mechanisms that have been well described for other autoantigens 44. Future studies will have to identify the mechanisms that prevent this repertoire purging in patients with NMO. Interestingly, the coincidence of NMO and myasthenia gravis—a peripheral autoimmune disease mediated by autoantibodies to the α‐subunit of the acetylcholine receptor—is higher than expected by chance 45, and it is intriguing to speculate that a general defect in B‐cell tolerance might be a common denominator.

In summary, the major immunogenic and also encephalitogenic T‐cell epitope in the context of IA^b^ is AQP4(201–220). However, a tight deletional tolerance precludes the development of an AQP4‐directed panencephalitis upon sensitization with AQP4 in WT mice. In addition, a serum response to AQP4 is not elicited in WT mice, not because of insufficient T‐cell help but because the B‐cell repertoire is also purged of AQP4‐specific B cells. Thus, after advancing our understanding of the effector phase of NMO, we need to obtain a more detailed understanding of the mechanisms that lead to the breakdown of central and peripheral tolerance in the T‐cell and B‐cell compartment of patients with NMO. With the knowledge on the relevant epitopes, the investigation of potential molecular events in a homologous mouse system might be a feasible approach.

## Materials and methods

### Mice

C57BL/6 mice, *Tcra* ^−/−^ mice, and *Rag1* ^−/−^ mice were obtained from Jackson Laboratories. *Aqp4* ^−/−^ mice were kindly provided by A. Verkman (University of California, San Francisco UCSF) and have been described before 24. All mice were housed in a pathogen‐free facility at the Technical University of Munich. All experimental protocols were approved by the standing committee for experimentation with laboratory animals of the Bavarian or Rhine Palatinate state authorities and carried out in accordance with the corresponding guidelines (AZ 55.2‐1‐54‐2532‐29‐13 and 55.2‐1‐54‐2532‐95‐2014).

### Antigens

Mouse MOG(35–55), MEVGWYRSPFSRVVHLYRNGK, and all mouse AQP4 peptides spanning the whole AQP4 sequence overlapping by 15 amino acids including AQP4(201–220), HLFAINYTGASMNPARSFGP (see Supporting Information Tables), were synthesized by Auspep (Tullamarine, Australia) or Biotrend (Cologne, Germany), respectively. rMOG protein was obtained from Biotrend and mouse AQP4 protein was produced using a baculovirus‐insect cell expression system and purified as described below.

### AQP4 protein expression and purification

The full‐length mouse *Aqp4* gene (NCBI accession no. NM_009700.2) was amplified via PCR of cDNA from mouse cerebellum (Clontech). The expression construct was designed with a C‐terminal 6xHIS tag and cloned into the *Xba*I and *Hin*dIII sites of pFBDM expression vector. The resulting vector was introduced into MultiBac baculoviral DNA in DH10MultiBac *Escherichia coli* cells. Bacmid DNA was prepared from selected clones and further used to transfect insect cells for protein production. Infected High Five cell cultures were grown at a 6 l spinning flask format for 48 h before being harvested by centrifugation at 4°C at 6000 × *g* for 10 min. Pelleted cells were stored at –20°C. Purification of the AQP4 protein was performed according to 46. First, cells were thawed and washed twice in TBS buffer with 1 mM β‐mercaptoethanol (ME). Then, cells were resuspended in the same buffer and lysed by two ultrasonication cycles of 30 sec each. Unlysed cells were removed by centrifugation at 4°C at 6000 × *g* for 10 min. The membranes were pelleted by ultracentrifugation at 4°C at 160 000 × *g* for 1 h and resuspended in 25 mL MR buffer (25 mM Tris, 250 mM NaCl, 10% glycerol, 1 mM β‐ME, pH 7.4 at room temperature). To keep membrane proteins in solution, 25 ml MR buffer with additional 400 mM *n*‐octyl‐β‐d‐glucopyranoside was added to the cell mixture and stirred at 4°C for 1 h. With a second ultracentrifugation at 4°C at 160 000 × *g* for 30 min unsolubilized material was pelleted and removed. Supernatants were incubated with Ni‐NTA beads at 4°C for 1 h. After washing the Ni‐NTA beads two times with MR buffer containing 40 mM *n*‐octyl‐β‐d‐glucopyranoside and 25 mM Imidazole, Ni‐NTA‐bound proteins were eluted with 250 mM Imidazole. AQP4 was concentrated using 30 000 molecular weight cut‐off Amicon spin concentrator (Millipore) and further purified via gel filtration on a S200 HR 16/60 column. Fractions of the peaks were pooled and once again concentrated using an Amicon spin concentrator. The final yield of AQP4 was up to 1 mg of purified protein per liter of cells.

### Immunization procedures and induction of disease

Mice were immunized subcutaneously at the base of tail with 200 μL of an emulsion containing either 200 μg of peptide or 100 μg of protein antigen and 250 μg *Mycobacterium tuberculosis* H37Ra (BD Difco) in mineral oil (CFA). In addition, mice received 200 ng pertussis toxin (Ptx, Sigma) i.v. on the days 0 and 2 after immunization. Where indicated, serum transfer was performed i.v. on days 6 and 12 after immunization using either 100 μL of naive or immune serum. The latter was harvested from AQP4‐immunized *Aqp4* ^−/−^ mice and showed high titers of anti‐AQP4 antibodies. Alternatively, mice received i.v. injections of 20 μg of a monoclonal antibody recognizing mouse AQP4 (rAb‐53). Murine anti‐AQP4 antibody (rAb‐53) is a human–mouse chimeric recombinant antibody (rAb) generated by replacing the human IgG1 Fc region of an AQP4‐specific, NMO patient derived CSF plasma cell clone 12 with a mouse IgG2a Fc region. Control rAb IC‐05 is a divalent human IgG1 antibody of unknown specificity derived from a chronic meningitis patient 47. Clinical signs of disease were monitored daily with scores as follows: 0, no disease; 1, loss of tail tone; 2, impaired righting; 3, paralysis of both hind limbs; 4, tetraplegia; 5, moribund.

### Generation of BM chimeras

For the generation of BM chimeras, recipient mice were lethally irradiated. The 7 gray (Gy) total dose was delivered as two 3.5 Gy doses three hours apart. A total of 5 × 10^6^ BM cells were injected i.v. into recipients 1 day after irradiation. Reconstituted mice were maintained on antibiotic‐water (Enrofloxacin, Bayer, 0.1 mg/mL) for 2 weeks after cell transfer. Reconstitution of the hematopoietic system was tested in the peripheral blood.

### Generation of compound mice

Mature CD4^+^ T cells were isolated from unmanipulated *Aqp4* ^−/−^ mice by using CD4 untouched beads (Miltenyi Biotec), and 14 × 10^6^ cells were transferred intraperitoneally (i.p.) into *Tcra* ^−/−^ or *Rag1* ^−/−^ mice to create *Aqp4* ^ΔT^ × *Tcra* ^−/−^ and *Aqp4* ^ΔT^ × *Rag1* ^−/−^ mice, respectively. One day after transfer, these compound mice were immunized as indicated.

### Proliferation assays

After immunization with full‐length AQP4 protein, mice were sacrificed on day 12 after immunization and draining LN cells and splenocytes were harvested to perform peptide scan analysis. A total of 350 000 cells were plated in a 96‐well plate format together with single 20‐mer peptides with a final concentration of 10 μg/mL in clone medium (DMEM‐10% FCS supplemented with 5 × 10^−5^ M β‐ME, 1 mM sodium pyruvate, nonessential amino acids, l‐glutamine and 100 U penicillin/100 μg streptomycin per milliliter). Cells were cultured for 72 h at 37°C and 5% CO_2_ and were pulsed with 1 μCi of ^3^[H] thymidine (PerkinElmer) for the last 16 h. To measure the proliferation rate, cells were harvested on glass fiber filters and analysis of incorporated ^3^[H] thymidine was performed in a β‐counter (1450 Microbeta; Trilux, PerkinElmer).

### Flow cytometric assay to detect conformational anti‐AQP4 and anti‐MOG antibodies

To detect anti‐AQP4 or anti‐MOG antibodies in the collected sera of protein immunized mice, a cell‐based flow cytometric assay was used that has been described before 25. Sera were diluted (1:50) in Roswell Park Memorial Institute (RPMI) 1640 growth medium and added to a 96‐well plate that contained 30 000 LN18^AQP4^ or LN18^MOG^ cells per well. As control every serum was tested on LN18^CTRL^ cells (transduced with empty vector). The plate was incubated on ice on an orbital shaker for 25 min. Cells were washed twice with fluorescence‐activated cell sorting (FACS) buffer (2% FCS in phosphate‐buffered saline [PBS]). To stain murine antibodies, 50 μL of diluted (1:100 in washing buffer) AlexaFluor488‐labeled goat anti‐mouse IgG H+L (Life Technologies, Thermo Scientific) were added to each well. After incubation for 25 min on ice on an orbital shaker, the cells were washed twice with FACS buffer. Cell surface staining was analyzed using a CyAnADP 9 flow cytometer (Beckman/Coulter) and FlowJo software (Tree Star).

### OCT analysis

OCT examination was performed by a common spectral domain OCT with TruTrack Eye Tracking technology (Spectralis, Heidelberg Engineering, Germany) as previously described 48. Briefly, for adaptation to murine eyes, a 78‐diopter lens (double aspheric Volk lens 78D; Volk Optical Inc., Mentor, USA) was placed directly in front of the OCT machine. Mice were anesthetized by i.p. application of medetomidine (0.5 mg/kg), midazolam (5 mg/kg), and fentanyl (0.05 mg/kg), wore a 100‐diopter contact lens (Roland Consult, Germany) with contact gel, and were placed on a self‐manufactured platform in front of the OCT machine. Eyes were treated with eye drops containing 2% atropine before the procedure. The retina was analyzed using a volume scan centered on the optic nerve head consisting of 12 vertical B‐scans (each with 768 A scans) with a scanning angle of 15° × 15°. Both eyes were measured and each scan was assessed for sufficient signal strength (>15 dB), adequate illumination, and accurate beam placement. Only scans that met these quality criteria were used for analysis. Every B‐scan was segmented automatically into different layers using the Eye Explorer version 6.0.9.0 provided by Heidelberg Engineering. Segmentations were checked manually and corrected if necessary in a blinded manner. Here, the optic nerve head was identified through opening of the Bruch's membrane and excluded for volumetric analyses. For longitudinal measurements, a company‐derived “follow‐up” software mode was used, which enabled repetitive analyses of the exact same place.

### Histology

Mice were sacrificed under deep anesthesia by intracardial perfusion with PBS followed by perfusion with 4% w/v paraformaldehyde solved in PBS. Brains, spinal cords, optic nerves, kidneys and spleens were removed and fixed in 4% paraformaldehyde overnight. Cervical, thoracic, and lumbar spinal cord was cut into 11–12 4 mm thick transverse segments prior to embedding; 5 μm thick sections were stained for hematoxylin and eosin (H&E) and Luxol‐fast blue/periodic acid‐Schiff. Immunohistochemistry was performed using a biotin‐streptavidin peroxidase technique (K5001; Dako) and an automated immunostainer (AutostainerLink 48; Dako). Sections were pretreated in a steamer (treatment solutions pH 6.0 or 9.0; Dako) before incubation with the primary antibodies anti‐Mac3 (clone M3/84, 553322, 1:100; BD Pharmingen), anti‐CD3 (MCA 1477, 1:50; Serotec), and anti‐AQP4 (HPA014784, 1: 4000; Sigma). DAB was used as a chromogen and sections were counterstained using hematoxylin.

To quantify the perivascular loss of immunoreactivity against AQP4, three consecutive brain sections per mouse were analyzed using Image J (NIH) 49. The sizes of areas showing AQP4 loss and the corresponding vessel lumen were measured and processed as individual ratios (any ratio > 1 indicates perivascular AQP4 loss).

### Statistical analysis

Statistical evaluations of cell numbers, MFIs, and OCT data were performed with the unpaired Student's *t*‐test when two populations were compared. Two‐tailed *p* values < 0.05 were considered significant. Multiple comparisons were performed with one‐way ANOVA followed by specific post‐tests as indicated in the legends of the figures. Clinical scores between groups were analyzed as disease burden using a Mann–Whitney *U* test for nonparametric values.

## Conflict of interest

The authors declare no financial or commercial conflict of interest.

**Abbreviations**
AQP4: aquaporin 4
INL: inner nuclear layer
MOG: myelin oligodendrocyte glycoprotein
NMO: neuromyelitis optica
OCT: optical coherence tomography

## Supporting information

Peer review correspondence

Supporting Information
