# Supplementary material for: Deletional tolerance prevents AQP4‐directed autoimmunity in mice
Source: Eur J Immunol. 2017 Jan 25;47(3):458–69. doi: 10.1002/eji.201646855 (PMC5359142; doi:10.1002/eji.201646855)
Supplement: Supplementary file 2 — Supporting Information [file EJI-47-458-s002.pdf]

European Journal of Immunology

Supporting Information
for
DOI 10.1002/eji.201646855

Anna-Lena Vogel, Benjamin Knier, Katja Lammens, Sudhakar Reddy Kalluri, Tanja Kuhlmann, Jeffrey L. Bennett and Thomas Korn

Deletional tolerance prevents AQP4-directed autoimmunity in mice

© 2017 The Authors. *European Journal of Immunology* published by WILEY-VCH Verlag GmbH & Co. KGaA, Weinheim.

Supporting Information

Supplementary Figure 1

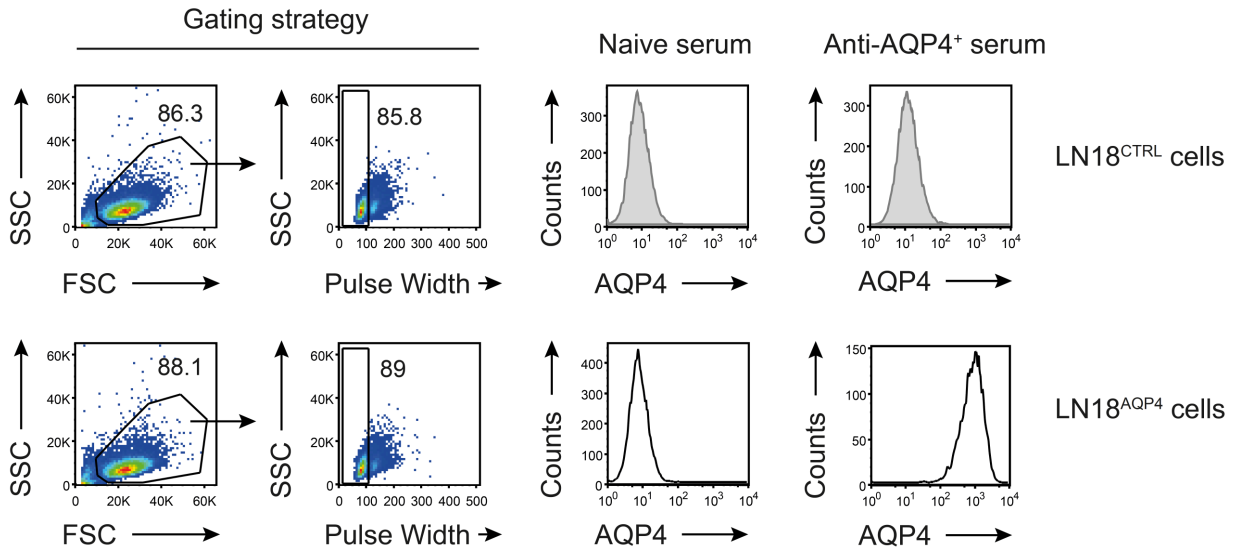

Supplementary Figure 1. Cell based assay to measure conformational anti-AQP4 antibodies in sera of mice. LN18 cells were either transduced with empty vector ($LN18^{CTRL}$ cells, top row) or with an AQP4 expressing lentivirus ($LN18^{AQP4}$ cells, bottom row). $LN18^{CTRL}$ or $LN18^{AQP4}$ cells were incubated with naive serum or anti-$AQP4^{+}$ serum as described in Materials and Methods. Anti-mouse total IgG H+L (AlexaFluor488 labeled) was used to detect anti-AQP4 antibodies bound to $LN18^{CTRL}$ cells (shaded histograms) or $LN18^{AQP4}$ cells (open histograms). Gating strategy and representative histogram plots. Numbers indicate percentage fractions of parental populations in the respective gates.

Supplementary Table 1

AQP4 peptides (20-mers) overlapping by 15 amino acids and spanning the whole sequence of mouse AQP4 protein.

Mouse AQP4 Peptides (20-mers)		
Peptide No	AA residues	Mouse 20-mer Peptides (Sequence)
P-01	mAQP4_1-20	MSDRAAARRWGKCGHSCSRE
P-02	mAQP4_6-25	AARRWGKCGHSCSRESIMVA
P-03	mAQP4_11-30	GKCGHSCSRESIMVAFKGVW
P-04	mAQP4_16-35	SCSRESIMVAFKGVWTQAFW
P-05	mAQP4_21-40	SIMVAFKGVWTQAFWKAVSA
P-06	mAQP4_26-45	FKGVWTQAFWKAVSAEFLAT
P-07	mAQP4_31-50	TQAFWKAVSAEFLATLIFVL
P-08	mAQP4_36-55	KAVSAEFLATLIFVLLGVGS
P-09	mAQP4_41-60	EFLATLIFVLLGVGSTINWG
P-10	mAQP4_46-65	LIFVLLGVGSTINWGGSENP
P-11	mAQP4_51-70	LGVGSTINWGGSENPLPVDM
P-12	mAQP4_56-75	TINWGGSENPLPVDMVLISL
P-13	mAQP4_61-80	GSENPLPVDMVLISLCFGLS
P-14	mAQP4_66-85	LPVDMVLISLCFGLSIATMV
P-15	mAQP4_71-90	VLISLCFGLSIATMVQCFGH
P-16	mAQP4_76-95	CFGLSIATMVQCFGHISGGH
P-17	mAQP4_81-100	IATMVQCFGHISGGHINPAV
P-18	mAQP4_86-105	QCFGHISGGHINPAVTVAMV
P-19	mAQP4_91-110	ISGGHINPAVTVAMVCTRKI
P-20	mAQP4_96-115	INPAVTVAMVCTRKISIAKS
P-21	mAQP4_100-120	TVAMVCTRKISIAKSVFYII
P-22	mAQP4_106-125	CTRKISIAKSVFYIIAQCLG
P-23	mAQP4_111-130	SIAKSVFYIIAQCLGAIIGA
P-24	mAQP4_116-135	VFYIIAQCLGAIIGAGILYL
P-25	mAQP4_121-140	AQCLGAIIGAGILYLVTPPS
P-26	mAQP4_126-145	AIIGAGILYLVTPPSVVGGL
P-27	mAQP4_131-150	GILYLVTPPSVVGGLGVTTV
P-28	mAQP4_136-155	VTPPSVVGGLGVTTVHGNLT
P-29	mAQP4_141-160	VVGGLGVTTVHGNLTAGHGL
P-30	mAQP4_146-165	GVTTVHGNLTAGHGLLVELI

P-31	mAQP4_151-170	HGNLTAGHGLLVELIITFQL
P-32	mAQP4_156-175	AGHGLLVELIITFQLVFTIF
P-33	mAQP4_161-180	LVELIITFQLVFTIFASCDS
P-34	mAQP4_166-185	ITFQLVFTIFASCDSKRTDV
P-35	mAQP4_171-190	VFTIFASCDSKRTDVTGSIA
P-36	mAQP4_176-195	ASCDSKRTDVTGSIALAIGF
P-37	mAQP4_181-200	KRTDVTGSIALAIGFSVAIG
P-38	mAQP4_186-205	TGSIALAIGFSVAIGHLFAI
P-39	mAQP4_191-210	LAIGFSVAIGHLFAINYTGA
P-40	mAQP4_196-215	SVAIGHLFAINYTGASMNPA
P-41	mAQP4_201-220	HLFAINYTGASMNPARSFGP
P-42	mAQP4_206-225	NYTGASMNPARSFGPAVIMG
P-43	mAQP4_211-230	SMNPARSFGPAVIMGNWANH
P-44	mAQP4_216-235	RSFGPAVIMGNWANHWIYWV
P-45	mAQP4_221-240	AVIMGNWANHWIYWVGPIMG
P-46	mAQP4_226-245	NWANHWIYWVGPIMGAVLAG
P-47	mAQP4_231-250	WIYWVGPIMGAVLAGALYEY
P-48	mAQP4_236-255	GPIMGAVLAGALYEYVFCPD
P-49	mAQP4_241-260	AVLAGALYEYVFCPDVELKR
P-50	mAQP4_246-265	ALYEYVFCPDVELKRRLKEA
P-51	mAQP4_251-270	VFCPDVELKRRLKEAFSKAA
P-52	mAQP4_256-275	VELKRRLKEAFSKAAQQTKG
P-53	mAQP4_261-280	RLKEAFSKAAQQTKGSYMEV
P-54	mAQP4_266-285	FSKAAQQTKGSYMEVEDNRS
P-55	mAQP4_271-290	QQTKGSYMEVEDNRSQVETE
P-56	mAQP4_276-295	SYMEVEDNRSQVETEDLILK
P-57	mAQP4_281-300	EDNRSQVETEDLILKPGVVH
P-58	mAQP4_286-305	QVETEDLILKPGVVHVIDID
P-59	mAQP4_291-310	DLILKPGVVHVIDIDRGEEK
P-60	mAQP4_296-315	PGVVHVIDIDRGEEKKGKDS
P-61	mAQP4_301-320	VIDIDRGEEKKGKDSSGEVL
P-62	mAQP4_304-323	IDRGEEKKGKDSSGEVLSSV

Supplementary Table 2

AQP4 peptides (11-mers) overlapping by 10 amino acids and spanning the sequence of mouse AQP4(196-225).

Mouse AQP4 "P41" region fine mapping (11-mers)		
Peptide No	AA residues	Mouse 11-mer Peptides (Sequence)
P41-01	mAQP4_196-206	SVAIGHLFAIN
P41-02	mAQP4_197-207	VAIGHLFAINY
P41-03	mAQP4_198-208	AIGHLFAINYT
P41-04	mAQP4_199-209	IGHLFAINYTG
P41-05	mAQP4_200-210	GHLFAINYTGA
P41-06	mAQP4_201-2011	HLFAINYTGAS
P41-07	mAQP4_202-212	LFAINYTGASM
P41-08	mAQP4_203-213	FAINYTGASMN
P41-09	mAQP4_204-214	AINYTGASMNP
P41-10	mAQP4_205-215	INYTGASMNPA
P41-11	mAQP4_206-216	NYTGASMNPAR
P41-12	mAQP4_207-217	YTGASMNPARS
P41-13	mAQP4_208-218	TGASMNPARSF
P41-14	mAQP4_209-219	GASMNPARSFG
P41-15	mAQP4_210-220	ASMNPARSFGP
P41-16	mAQP4_211-221	SMNPARSFGPA
P41-17	mAQP4_212-222	MNPARSFGPAV
P41-18	mAQP4_213-223	NPARSFGPAVI
P41-19	mAQP4_214-224	PARSFGPAVIM
P41-20	mAQP4_215-225	ARSFGPAVIMG
