# Supplementary material for: Deletional tolerance prevents AQP4‐directed autoimmunity in mice
Source: Eur J Immunol. 2017 Jan 25;47(3):458–69. doi: 10.1002/eji.201646855 (PMC5359142; doi:10.1002/eji.201646855)
Supplement: Supplementary file 1 — Peer review correspondence [file EJI-47-458-s001.pdf]

European Journal of Immunology

Deletional tolerance prevents AQP4 directed autoimmunity in mice

Anna-Lena Vogel, Benjamin Knier, Katja Lammens, Sudhakar Reddy Kalluri, Tanja Kuhlmann, Jeffrey L. Bennett and Thomas Korn

Correspondence: Dr. Thomas Korn, Department of Neurology, Klinikum rechts der Isar, Munich, Germany

Review Timeline:	Submission date:	6-Dec-2016
	First Editorial decision:	19-Dec-2016
	First Revision received:	22-Dec-2016
	Accepted:	2-Jan-2017

Handling Executive Committee member: Dr. Steffen Jung

Please note that the correspondence below does not include the standard editorial instructions regarding preparation and submission of revised manuscripts, only the scientific revisions requested and addressed.

First Editorial Decision -19-Dec-2016

Dear Dr. Korn,

Manuscript ID eji.201646855 entitled "Deletional tolerance prevents AQP4 directed autoimmunity in mice", which you submitted to the European Journal of Immunology, has been reviewed. The comments of the referees are included at the bottom of this letter.

Although the referees have recommended publication, some revisions to your manuscript have been requested. Therefore, I invite you to respond to the comments of the referees and revise your manuscript accordingly.

You should also pay close attention to the editorial comments included below. **In particular, please edit your figure legends to follow Journal standards as outlined in the editorial comments. Failure to do this will result in delays in the re-review process.**

If the revision of the paper is expected to take more than three months, please inform the editorial office. Revisions taking longer than six months may be assessed by new referee(s) to ensure the relevance and

timeliness of the data.

Once again, thank you for submitting your manuscript to European Journal of Immunology. We look forward to receiving your revision.

Yours sincerely,
Laura Soto Vazquez

on behalf of Dr. Steffen Jung

Editorial Office
European Journal of Immunology
e-mail: ejied@wiley.com
www.eji-journal.eu

****

Reviewer: 1

Comments to the Author

In this work Vogel et al investigate the mechanisms that control the pathogenic autoimmune response to aquaporin 4 (AQP4).   Antibodies reactive with AQP4 in astrocytes are thought to mediate disease pathogenesis in neuromyelitis optica (NMO), however the mechanisms that control their generation are mostly unknown. Using AQP4-deficient mice, Vogel et al identified an immunodominant epitope in AQP4201-220. In addition, they found that AQP4 specific T cell and B cell responses are purged from the mature immune receptor repertoire, probably as a result of the expression of AQP4 in thymic stromal cells and other peripheral tissues that enforce deletional T and B cell tolerance.

Strikingly, the authors also found that AQP4201-220-specific T cells induce a neurologic syndrome when transferred into mice; this syndrome can be amplified by the transfer of AQP4-specific antibodies. Moreover, co-transfer of AQP4 specific T cells and antibodies results in the generation of lesions that show a distribution patter similar to those that characterize NMO. Finally, co-transfer of AQP4 reactive T cells and antibodies resulted in retinal lesions detectable by optical coherence tomography. Collectively, this work characterizes the immunological mechanisms that control the pathologic immune response directed against AQP4. In addition, these studies describe a new experimental model where to study NMO immune pathology and potential therapeutic interventions.

The experiments are extremely well designed, with appropriate controls included. In addition, the results are properly described and the conclusions are supported by the data presented. This work should be of interest to the broad readership of EJI. I only have minor comments that may improve the quality of this work.

Fig. 1. Please indicate size of full-length AQP4 in the gels with an arrow.
Fig. 4.a. Please clarify the text to state that the bone marrow chimeras were immunized with AQP4.
Fig. 4.b. Please indicate when anti-CD25 was administered.
Fig. 5e. Please include a bar to indicate the scale in these figures.

Reviewer: 2

Comments to the Author

The authors immunized AQP4-/- mice with AQP4 and identified AQP4(201-220) as major immunogenic IAb epitope of AQP4. They also show that this T cell reactivity is deleted in AQP4+/+ mice. In addition, the authors could also show that the B cell repertoire of wild type mice is "purged"• of AQP4 specific B cells.

In all, this is an interesting paper, with well conducted experiments.

1. The description of their findings needs some improvement, as can be seen in a number of examples:
- on p. 10, Results: they state that "AQP4(201-220) T cells induce a clinically manifest encephalomyelitis"¦."• . Here, the statement that this is "most likely due to an astrocyte disease"• is wrong and should be deleted.
- on p.10, Results: they write, "NMO-specific lesional patterns in the CNS are only achieved with anti-AQP4 antibodies."• This is not true: NMO-specific lesional patterns in the CNS are only achieved in the presence of AQP4 (201-220) specific T cells and anti-AQP4 antibodies.
- on p.10, Discussion: "NMO-like lesions in the retina and in the CNS, which are characterized by perivascular loss of AQP4 reactivity, are only induced in the presence of anti-AQP4 antibodies, but not by AQP4(201-220) specific T cells alone"• . The authors never show the presence of NMO-like lesions in the retina, but only used OCT. Moreover, they also did not show the action of rAB53 on retina and CNS inflammation alone in their results. Since it is clear from the literature that the antibodies alone do not make any damage, I do not insist on showing such results. However, the authors should clearly write: NMO-like lesions in the CNS"¦.. are only induced in the presence of anti-AQP4 antibodies AND AQP4(201-230) SPECIFIC T CELLS, but not by AQP4(201-220) specific T cells alone"• .
- on p.11: the authors write that after transfer of low numbers of activated MBP specific T cell blasts together

with human IgG reactive to AQP4 into Lewis rats"¦., "clinical signs of disease do not develop in this model"• . This is wrong, since in all MBP-T cell-based ENMO studies published so far, T cell numbers sufficed to produce clinical symptoms.
-on p. 12: "Our findings that INL swelling as measured by retinal OCT only occurred in the presence of anti-AQP4 antibodies"¦"• this has to be corrected to ""¦.only occurred in the presence of both anti-AQP4 antibodies and AQP4-specific T cells."•

2. the authors only identified AQP4(201-220) as immunogenic epitope in their AQP4-/- C57BL/6 mice. This is in contrast to a recently published study (Sagan et al., PNAS, doi: 10:10.1073/pnas.1617859114) where the authors identified AQP4 (201-230), but also identified AQP4(135-153). The authors should discuss this discrepancy.

3. The authors did not show any data from bone marrow chimeras. Therefore, they should delete this part from materials and methods.

First Revision – authors' response 23-Dec-2016

Reviewer #1:
We would like to thank this reviewer for his/her very positive assessment.

Fig. 1. Please indicate size of full-length AQP4 in the gels with an arrow.
Fig. 4.a. Please clarify the text to state that the bone marrow chimeras were immunized with AQP4.
Fig. 4.b. Please indicate when anti-CD25 was administered.
Fig. 5e. Please include a bar to indicate the scale in these figures.
Response: All these points were fixed in the revised version of the manuscript.

Reviewer #2:
We are grateful to this reviewer for providing very constructive suggestions that helped to improve the quality of our manuscript.

1. The description of their findings needs some improvement, as can be seen in a number of examples:
- on p. 10, Results: they state that "AQP4(201-220) T cells induce a clinically manifest encephalomyelitis….". Here, the statement that this is "most likely due to an astrocyte disease" is wrong and should be deleted.

Response: We removed this statement in the revised version of the manuscript, as suggested.

- on p.10, Results: they write, "NMO-specific lesional patterns in the CNS are only achieved with anti-AQP4 antibodies." This is not true: NMO-specific lesional patterns in the CNS are only achieved in the presence of AQP4 (201-220) specific T cells and anti-AQP4 antibodies.

Response: We apologize for having been unclear in this paragraph. Of course, it was not our intention to imply that NMO like lesions can be induced by anti-AQP4 antibodies only. In order to clarify this paragraph in the revised version of our manuscript, it now reads:
"Taken together, AQP4(201-220)-specific T cells alone induce a clinically manifest encephalomyelitis. However, NMO-specific lesional patterns in the CNS are only achieved in the additional presence of anti-AQP4 antibodies." (p. 10, revised manuscript)

- on p.10, Discussion: "NMO-like lesions in the retina and in the CNS, which are characterized by perivascular loss of AQP4 reactivity, are only induced in the presence of anti-AQP4 antibodies, but not by AQP4(201-220) specific T cells alone". The authors never show the presence of NMO-like lesions in the retina, but only used OCT. Moreover, they also did not show the action of rAB53 on retina and CNS inflammation alone in their results. Since it is clear from the literature that the antibodies alone do not make any damage, I do not insist on showing such results. However, the authors should clearly write: NMO-like lesions in the CNS….. are only induced in the presence of anti-AQP4 antibodies AND AQP4(201-230) SPECIFIC T CELLS, but not by AQP4(201-220) specific T cells alone" .

Response: According to the reviewer's suggestion, we tuned down our statements on retinal pathology since we indeed do not show retinal histology. In addition, we made the requirement for antigen-specific T cells and antibodies very clear by re-phrasing this paragraph (p. 11, revised manuscript):
"Despite the encephalitogenic potential of AQP4(201-220)-specific T cells, NMO-like lesions in the CNS, which are characterized by perivascular loss of AQP4 immunoreactivity, are only induced in the presence of AQP4(201-220)-specific T cells and anti-AQP4 antibodies but not by AQP4(201-220)-specific T cells alone."

- on p.11: the authors write that after transfer of low numbers of activated MBP specific T cell blasts together with human IgG reactive to AQP4 into Lewis rats…., "clinical signs of disease do not develop in this model". This is wrong, since in all MBP-T cell-based ENMO studies published so far, T cell numbers sufficed to produce clinical symptoms.

Response: We apologize for this wrong statement and revised the relevant paragraph accordingly: "Early work suggested that a mild experimental autoimmune encephalomyelitis induced by adoptive transfer of encephalitogenic T cells of unrelated specificity (against myelin basic protein, MBP) is required to enable

access of i.v. transferred human anti-AQP4 antibodies to their target in the rat CNS [11],[12]. Thus, any T-cell mediated inflammation of the blood-brain-barrier appeared to be sufficient to enable anti-AQP4 antibody mediated tissue damage. However, a more meticulous investigation of the effector function of AQP4-specific T cells in situ has recently suggested that AQP4-specific T cells may determine the predilection sites that are typically affected by anti-AQP4 antibody mediated pathology [35]." (p. 11, revised discussion)

-on p. 12: “Our findings that INL swelling as measured by retinal OCT only occurred in the presence of anti-AQP4 antibodies…” this has to be corrected to “….only occurred in the presence of both anti-AQP4 antibodies and AQP4-specific T cells.”

Response: This is now corrected in the revised version: " Our finding that INL swelling as measured by retinal OCT only occurred in the presence of both AQP4-specific T cells and anti-AQP4 antibodies but not in T cell mediated encephalomyelitis with either AQP4 or MOG as target antigens alone is in line with a loss-of-function of Müller cells, which play an important role in water and potassium homeostasis of the INL of the retina [37]." (p. 12 of the revised manuscript)

2. the authors only identified AQP4(201-220) as immunogenic epitope in their AQP4-/- C57BL/6 mice. This is in contrast to a recently published study (Sagan et al., PNAS, doi: 10:10.1073/pnas.1617859114) where the authors identified AQP4 (201-230), but also identified AQP4(135-153). The authors should discuss this discrepancy.

Response: We are quite confident that this second epitope, AQP4(135-153) is not a naturally occurring epitope of AQP4. In the Sagan paper, the authors did not use full length AQP4 protein to immunize Aqp4-/- mice, which we did, but used an "in silico" approach and selected good IAb binders as potential AQP4 epitopes. The fact that adoptive transfer of highly activated AQP4(135-153) specific T cells produces transient symptoms in recipient mice is not an appropriate experiment to confirm a natural epitope. Active immunization with that epitope would be; and here, no clinical signs of disease are reported (Sagan et al., 2016).
We now discuss this problem in the text of the discussion on p. 13 of the revised manuscript: "Besides AQP4(201-220), a further IAb-restricted epitope of AQP4, i. e. AQP4(135-153), has been reported in the repertoire of Aqp4-/- mice [41],[43]. However, since AQP4(135-153) was inferred as a relevant epitope by using an "in silico" approach predicting binding affinities of peptides to IAb [43], it is possible that AQP4(135-153) may not be a naturally processed epitope of AQP4. Here, we did not find a significant AQP4(135-153)-specific recall response upon immunization of Aqp4-/- mice with full length murine AQP4 protein. However, we show that IAb-restricted AQP4(201-220) is indeed a naturally processed epitope of AQP4 and is able to induce an encephalomyelitic syndrome upon active immunization of Aqp4ΔT x Rag1-/-

mice."

3. The authors did not show any data from bone marrow chimeras. Therefore, they should delete this part from materials and methods.

Response: We indeed did show data from bone marrow chimeras (Fig. 4A). Thus, we would like to keep the description of the construction of these animals in the 'Materials and Methods' section.

Second Editorial Decision - 23-Dec-2016

Dear Dr. Korn,

It is a pleasure to provisionally accept your manuscript entitled "Deletional tolerance prevents AQP4 directed autoimmunity in mice" for publication in the European Journal of Immunology. For final acceptance, please follow the instructions below and return the requested items as soon as possible as we cannot process your manuscript further until all items listed below are dealt with.

Please note that EJI articles are now published online a few days after final acceptance (see Accepted Articles: http://onlinelibrary.wiley.com/journal/10.1002/(ISSN)1521-4141/accepted). The files used for the Accepted Articles are the final files and information supplied by you in Manuscript Central. You should therefore check that all the information (including author names) is correct as changes will NOT be permitted until the proofs stage.

We look forward to hearing from you and thank you for submitting your manuscript to the European Journal of Immunology.

Yours sincerely,
Laura Soto Vazquez

on behalf of Dr. Steffen Jung

Editorial Office
European Journal of Immunology
e-mail: ejied@wiley.com
www.eji-journal.eu
